# Effects of in-store marketing on food and beverage purchases: a longitudinal study of households with children

**DOI:** 10.1017/S1368980023002641

**Published:** 2023-12-01

**Authors:** Anna H Grummon, Joshua Petimar, Alyssa J Moran, Emma Anderson, Peter Lurie, Sara John, Eric B Rimm, Anne N Thorndike

**Affiliations:** 1 Department of Pediatrics, Stanford University School of Medicine, Palo Alto, CA 94304, USA; 2 Department of Health Policy, Stanford University School of Medicine, Stanford, CA, USA; 3 Department of Population Medicine, Harvard Medical School & Harvard Pilgrim Health Care Institute, Boston, USA; 4 Department of Health Policy & Management, Johns Hopkins Bloomberg School of Public Health, Baltimore, MD, USA; 5 Department of Population Health Management, Cambridge Health Alliance, Cambridge, USA; 6 Center for Science in the Public Interest, Washington, USA; 7 Department of Nutrition, Harvard TH Chan School of Public Health, Boston, USA; 8 Department of Epidemiology, Harvard TH Chan School of Public Health, Boston, USA; 9 Department of Medicine, Massachusetts General Hospital and Harvard Medical School, Boston, USA

**Keywords:** Food marketing, Grocery store, Food purchases, Parents, Primary prevention

## Abstract

**Objective:**

Most food retailers display foods in prominent locations as a marketing strategy (i.e. ‘placement promotions’). We examined the extent to which households with children change their food and beverage purchases in response to these promotions.

**Design:**

We analysed a novel dataset of all products promoted in two supermarkets from 2016 to 2017, including promotion dates and locations (e.g. aisle endcaps and front registers). We linked promotions to all purchases from the supermarkets from 2016 to 2017 by a cohort of households with children. We calculated the number of weekly promotions in each of thirteen food and beverage groups (e.g. bread; candy) and used fixed effects regressions to estimate associations between number of weekly promotions and households’ weekly food purchases, overall and by Supplemental Nutrition Assistance Program (SNAP) participation.

**Setting:**

Two large supermarkets in Maine, USA.

**Participants:**

Eight hundred and twenty-one households with children.

**Results:**

Most promotions (74 %) were for less healthy foods. The most promoted food groups were sweet and salty snacks (mean = 131·0 promotions/week), baked goods (mean = 68·2) and sugar-sweetened beverages (mean = 41·6). Households generally did not change their food group purchases during weeks when they were exposed to more promotions for those groups, except that a 1-sd increase in endcap candy promotions (about 1 promotion/week) was associated with $0·19/week (about 14·5 %) increase in candy purchases among SNAP nonparticipants (adjusted *P* < 0·001).

**Conclusions:**

In-store placement promotions for food groups were generally not associated with purchases of promoted food groups, perhaps because exposure to unhealthy food marketing was consistently high. Substantial changes to in-store food marketing may be needed to promote healthier purchases.

Most children in the USA have poor dietary quality^(1)^, increasing their risk for short- and long-term negative health outcomes, including metabolic syndrome and obesity^(2)^. One important contributor to unhealthy diet among children is food marketing^(3–8)^. Although a large body of research has documented how food marketing affects children^(4–8)^, the influence of food marketing on parents’ and caregivers’ purchasing behaviour is less clear, as few studies of food marketing have examined this population^(9)^ and none have assessed behaviour. Understanding the effects of food marketing on parents’ behaviour is critical for promoting children’s health given that parents influence their children’s diets both through shaping what foods they have access to and by modelling dietary behaviours^(10)^.

One important and understudied setting in which to examine the effects of food marketing is grocery stores, where Americans acquire two-thirds of their daily calories^(11)^. A wide array of in-store food marketing activities occur in grocery stores. Prominent among these is the strategic placement of products in store locations that attract customers’ attention (e.g. at aisle endcaps or the front register)^(12,13)^. Despite the prevalence of in-store placement promotions, it remains largely unknown how these promotions affect individual- or household-level purchases, as almost all studies of placement promotions have examined store-level sales^(14–16)^. This makes it challenging to ascertain how promotions affect individual consumers’ purchase decisions, if at all. Another major gap is that most studies of in-store placement promotions have only examined how these promotions affect sales of the specific product or brand placed on promotion^(17–21)^. These studies cannot, however, address how promotions affect the overall composition of households’ purchases, an outcome that is likely more relevant to public health than purchases of any given item. For example, promoting a given product (e.g. Coca-Cola) may increase households’ purchases of that particular product without increasing total purchases of the food group to which that product belongs (e.g. no net effect on weekly sugar-sweetened beverage purchases). Without information on how marketing affects households’ net purchases of healthier and less healthy food groups, it is difficult to gauge the potential effects of in-store placement promotions on dietary quality or population health.

The effect of in-store food promotions specifically among households with children is also unknown. Parents may be especially susceptible to in-store food marketing if they shop with their children because such marketing could affect them directly and also encourage their children to repeatedly request marketed items. This suspectibility^(22–25)^ may be exacerbated in retail settings because in-store food promotions often target children by displaying promoted items at children’s eye level^(26,27)^ or by promoting foods using child-directed advertising^(28–30)^.

To address these gaps, this study linked longitudinal data on in-store placement promotions at two large supermarkets to household-level data on food and beverage purchases from those supermarkets made by a cohort of households with children. Our primary objectives were to characterise households’ exposure to in-store placement promotions in these stores and to examine associations between in-store placement promotions and households’ purchases of healthier and less healthy food and beverage groups. A recent study of store sales data found that the effects of in-store placement promotions may vary by product healthfulness and by household participation in the Supplemental Nutrition Assistance Program (SNAP)^(16)^, so we also conducted analyses stratifying by these variables.

## Methods

### Participants

This study examined food and beverage purchases made by households with children that participated in two prior intervention studies evaluating fruit and vegetable incentive and meal-bundling interventions^(31,32)^. As detailed elsewhere^(31–33)^, from 2015 to 2016, research staff enrolled a total of 905 shoppers from two large supermarkets located in low-income communities in Maine (300 shoppers were recruited from Store 1 and 605 from Store 2). Both supermarkets were part of the same chain that consists of approximately 180 stores in five states in the Northeast. Participants were eligible if they were aged 18 years or older, lived in a household with at least one child aged 18 years or younger, read and understood English, used the study store as their primary food shopping location, and were their households’ primary shopper.

### Procedures

After providing informed consent, participants completed a baseline survey (described below) and were enrolled in the supermarket’s loyalty card programme. Participants were given a study loyalty card, allowing tracking of their purchases over time. To encourage participants to shop at the study store and use their loyalty card, the card provided study participants with a 5 % discount on all purchases at the study store.

### Data

The supermarket chain provided data on all in-store placement promotions mandated by the chain’s corporate headquarters to be implemented at all stores in the chain from 10 January 2016–31 December 2017. Placement promotions included product displays, such as those at the checkout (‘front register’) and on the ends of aisles (‘endcaps’), with or without additional signage (e.g. ‘special deal’). A minority (24 %) of placement promotions also included a price promotion; however, > 90 % of these price promotions simply drew attention to the product’s price (e.g. ‘low prices’) without providing a discount. Promotions were implemented in 1-week intervals and could repeat for multiple weeks. The marketing data included information on the promoted item’s Universal Product Code (UPC) and product description as well as details about the required promotion, including the display location (e.g. aisle endcap, front register) and dates of promotion (see also Supplemental Methods). Of the 10 123 unique promotions in the initial dataset, we excluded 1598 (15·8 %) that were for non-food items and 540 (5·3 %) for items missing a product description. After exclusions, the data included 7985 unique promotions for 3869 unique UPCs.

We determined which promotions were implemented during each week in each study store using the corporate marketing plan and information on the physical attributes of the stores (e.g. number of aisles/front registers; see Supplemental Methods). Interviews with store managers and the chain’s marketing manager confirmed this approach was accurate and that stores followed the corporate marketing plan with a high degree of compliance.

The chain additionally provided transaction-level data on all purchases made in 2016–2017 across both study stores. The data included information on purchased items’ UPC, product description, date of purchase, price, price discount, and quantity purchased as well as the loyalty card number associated with the transaction, if any. The data also indicated whether the transaction was made online and whether any part of the transaction was paid for using a SNAP electronic benefit transfer card (‘SNAP EBT’). Purchases were linked to study participants using their loyalty card numbers; we excluded transactions not linked to participants’ loyalty card numbers.

We obtained data on the healthfulness of the products promoted and purchased in the stores from Guiding Stars, a shelf-tag nutrition labelling programme that rates products using a nutrition algorithm. Guiding Stars rates products as less healthy (0 stars) or as varying degrees of healthy (i.e. good (1 star), better (2 stars) or best (3 stars)) based on nutritional content (e.g. fibre, added sugar and wholegrains)^(34,35)^. During the study period, Guiding Stars did not rate beverages, non-food items or foods without product descriptions, so we did not assess healthfulness of these items. We linked product healthfulness data to marketing and transaction data using products’ UPC; we had healthfulness data for 89·8 % (82 364 out of 91 756) of food items promoted and 89·6 % (462 916 out of 516 672) of food items purchased (percentages exclude beverages, non-food items and items without product descriptions).

Finally, we obtained information on participants’ household and sociodemographic characteristics from the surveys participants completed at enrolment. The surveys included questions about age, race and ethnicity, household size, and number of children in the household.

### Measures

We categorised all promoted and purchased items into food groups (e.g. bread, candy and sugar-sweetened beverages), drawing on a previously developed food categorisation system^(32,36)^ (see online supplementary material, Supplemental Table 1). To capture responses to more highly marketed food groups, we focused specifically on food groups with ten or more promotions per week on average and beverage groups with five or more promotions per week on average. This resulted in analysis of eight food groups (baked goods; bread; candy; cereal; cold and frozen desserts; fruits, vegetables, beans, and nuts; packaged entrees and sides; and sweet and salty snacks) and five beverage groups (alcohol; juice; low-calorie beverages; sugar-sweetened beverages; and unsweetened beverages). These groups accounted for approximately 84 % of all promotions.

Our primary exposure variables were the total number of promotions in each food group during each week (e.g. the total number of promotions for sugar-sweetened beverages per week). Because in-store marketing interventions and policies have targeted endcaps and front registers in checkout aisles^(37–43)^, we also examined the number of promotions in each food group at endcaps and at the front registers specifically. Finally, we examined the number of promotions in each food group that were for healthier items (i.e. received at least a 1-star rating from Guiding Stars nutrition rating system) and for less healthy items (0-star rating from Guiding Stars), excluding products that were not rated.

Our primary outcomes were households’ purchases of food groups, assessed in US dollars per week. Weekly purchases were calculated by summing all items households purchased from that food group in a given week and adding back the dollar value of any discounts that were applied to the purchased products. Discounts were added back to the sale price because some promotions came with discounts and not adding discounts back could have resulted in an underestimate of the association of promotions with customer purchases. We additionally examined purchases of healthier and less healthy items (defined using Guiding Stars ratings) in each food group and overall. We chose to examine total purchases of food groups because this outcome is likely more relevant for public health outcomes than purchases of specific products. For example, it may matter more for public health the total amount of fruits a household buys, rather than whether they purchase blueberries versus strawberries.

Finally, we categorised households as participating in SNAP if they used SNAP EBT to pay for any purchases during the study period and as nonparticipants otherwise.

### Analysis

We analysed purchases made from 10 January 2016–31 December 2017 to match the dates for which we had marketing data, excluding items purchased outside the study period (*n* 16 141 items; 2 % of all items purchased), items purchased online (where in-store promotions would not be expected to exert effects; *n* 68 773 items; 9 % of all items purchased) and items purchased from the same supermarket chain, but outside the customer’s primary store (*n* 13 003, 2 % of all items purchased) (see online supplementary material, Supplemental Figure 1). Analyses also excluded households with fewer than 2 weeks of purchases (*n* 60; 6 % of households) and households without enrolment survey data (*n* 1; < 1 % of households). The analytic sample included 705 013 items purchased across 36 050 transactions made by 821 households.

To characterise households’ exposure to in-store marketing, we calculated the mean, standard deviation and interquartile range of the number of promotions in each food group each week, overall and by location. We additionally estimated the mean weekly proportion of these promotions that were for healthier foods.

Next, we examined associations between exposure to in-store marketing and households’ food and beverage purchases using linear regression models with household fixed effects. These models used within-household variation in exposure to in-store marketing to estimate the association between number of weekly placement promotions and households’ purchases, thereby controlling for all time-invariant household characteristics. For each food group, we regressed weekly purchases of that food group on number of promotions in that food group during that week, using three separate models for all promotions (i.e. regardless of location), for promotions at endcaps and for promotions at front registers. We standardised the exposure variables prior to analyses so that coefficients could be interpreted as the change in purchases associated with a 1-sd increase in promotions. All models controlled for calendar month (i.e. January–December), calendar year (2016 *v*. 2017), indicators for holiday weeks (weeks of Easter, Fourth of July, Halloween, Thanksgiving or Christmas), the average proportional discount applied to items purchased in that food group during that week, intervention period (for the interventions implemented during the prior studies), the interaction between intervention period and intervention arm, SNAP issuance weeks in Maine (i.e. 10–14th of each month), whether the household used SNAP benefits for any transaction that week, and the interaction between SNAP issuance weeks and use of SNAP for any transaction that week. Models estimated robust standard errors clustered at the household level. We additionally estimated associations stratifying by SNAP participation. We adjusted for multiple testing within families of outcomes defined by food group and promotion location (i.e. three tests per food group for analyses overall, among SNAP participants and among non-participants) by controlling the false discovery rate at *q* = 0·05 using the Benjamini–Hochberg method^(44)^. Finally, to assess whether associations differed for healthier *v*. less healthy foods, we examined associations stratifying by product healthfulness; these analyses did not examine beverages, which were not rated by Guiding Stars at the time of the study. These analyses also used the Benjamini–Hochberg method to adjust for multiple comparisons within food groups (i.e. two tests per food group for analyses among healthier and less healthy foods)^(44)^.

We report two-sided 95 % CI and adjusted *P*-values (i.e. q-values). Analyses were conducted in Stata MP version 17.1 (StataCorp LLC) in 2022–2023.

## Results

The majority of participants (i.e. shoppers recruited into the study) were female (62 %) and identified as non-Hispanic White (92 %) (Table 1). Most participants had one child (38 %) or two children (42 %); 19 % had three or more children. Nearly one-third (30 %) participated in SNAP during the study period. Mean weekly spending across all items purchased was $115·40 (sd = $98·42).

**Table 1:** Sample characteristics, *n* 821 primary shoppers with children

Characteristic	Mean or *n*	sd or %
Age		
18–29 years	143	18 %
30–39 years	342	42 %
40–49 years	252	31 %
50 years or older	69	9 %
Sex		
Male	310	38 %
Female	499	62 %
Race/Ethnicity		
Non-Hispanic White	744	92 %
Non-Hispanic Black	11	1 %
Hispanic or Latino(a)	11	1 %
American Indian or Alaskan Native	6	1 %
Asian or Pacific Islander	10	1 %
Another race or ethnicity	26	3 %
Household size		
2	71	9 %
3	225	28 %
4	301	38 %
5 or more	204	25 %
Number of children		
1	315	38 %
2	345	42 %
3 or more	159	19 %
Self-reported amount of shopping done in study store		
Very little	131	16 %
Less than half	136	17 %
Half	147	18 %
More than half but not all	124	15 %
Almost all	280	34 %
Household participated in SNAP during study	243	30 %
Household’s mean weekly spending on food groups ($)		
Foods		
Baked goods	$3·91	$5·99
Bread	$4·21	$5·07
Candy	$1·31	$3·30
Cereal	$1·83	$3·54
Cold and frozen desserts	$1·85	$3·65
Fruits, vegetables, beans and nuts	$18·35	$19·86
Packaged entrees and sides	$7·14	$10·47
Sweet and salty snacks	$6·84	$8·68
Beverages		
Alcohol	$3·66	$10·42
Juice	$1·31	$2·78
Low-calorie drinks	$0·59	$2·08
Sugar-sweetened beverages	$4·08	$6·69
Unsweetened drinks	$2·70	$4·79

SNAP, Supplemental Nutrition Assistance Program.

Missing data ranged from 0 to 2·5 %.

The stores had an average of 563·7 (sd = 73·1) total placement promotions for foods and beverages per week, with an average of 319·3 (sd = 33·5) promotions at endcaps and 94·2 (sd = 29·3) at the front register. The most promoted food groups during the study period were sweet and salty snacks (mean of 131·0 (sd = 15·9) promotions per store per week), baked goods (68·2 (16·3) promotions per week) and sugar-sweetened beverages (41·6 (12·3) promotions per week) (Table 2). The least promoted food and beverage groups were cereal (mean = 13·0 (sd = 5·4) promotions per week) and juice (6·8 (3·6) promotions per week). For all food and beverage groups except for the candy group and the fruits, vegetables, beans, and nuts group, the majority of placement promotions were at endcaps. Many food groups that are typically less healthful were consistently highly promoted, such that even the lower end (i.e. 25th percentile) of weekly promotions for that group included a substantial number of promotions. For example, the 25th percentile of weekly promotions for sweet and salty snacks was 118 promotions; for baked goods it was fifty-four promotions, and for sugar-sweetened beverages it was thirty-two promotions. Across all unique promotions for foods, 26 % were for healthier foods (based on Guiding Stars ratings) and 74 % were for less healthy foods.

**Table 2:** Characteristics of in-store placement promotions

	Promotions per week				
Food or beverage group	Mean	sd	IQR	Mean (sd) proportion of promotions for healthier foods	
				Mean	sd
Foods					
Sweet and salty snacks					
All promotions	131·0	15·9	118–141	33·1	5·7
Endcaps	82·4	9·3	76–92	35·9	6·1
Front register	22·4	8·0	18–29	24·3	20·2
Baked goods					
All promotions	68·2	16·3	54–79	1·1	1·1
Endcaps	36·6	10·8	28–44	0·3	1·2
Front register	13·7	5·3	12–18	1·4	2·9
Fruits, vegetables, beans and nuts					
All promotions	38·2	9·7	31–45	60·9	17·2
Endcaps	10·7	4·6	8–14	58·7	29·7
Front register	14·1	7·5	10–19	69·3	20·5
Bread					
All promotions	32·9	10·2	25–41	46·3	12·5
Endcaps	23·4	6·8	18–28	51·6	12·3
Front register	1·5	1·7	0–3	0·0	0·0
Packaged entrees and sides					
All promotions	30·8	9·1	24–37	12·1	8·1
Endcaps	21·7	8·0	15–29	10·8	10·8
Front register	2·6	2·0	1–4	2·7	9·2
Cold and frozen desserts					
All promotions	17·4	7·1	13–22	2·4	5·0
Endcaps	17·2	6·9	13–21	2·4	4·9
Front register	0·0	0·0	0–0	–	–
Candy					
All promotions	17·2	10·3	9–24	0·0	0·0
Endcaps	0·5	1·3	0–0	0·0	0·0
Front register	12·7	9·4	6–19	0·0	0·0
Cereal					
All promotions	13·0	5·4	9–15	57·8	18·8
Endcaps	9·0	4·3	6–10	61·4	19·9
Front register	1·7	2·1	0–3	31·1	26·7
Beverages					
Sugar-sweetened beverages					
All promotions	41·6	12·3	32–48	–	–
Endcaps	29·1	8·0	23–36	–	–
Front register	3·3	3·6	0–5	–	–
Unsweetened drinks					
All promotions	30·0	7·2	24–34	–	–
Endcaps	17·3	3·5	15–19	–	–
Front register	2·0	2·3	0–3	–	–
Alcohol					
All promotions	27·2	16·7	12–40	–	–
Endcaps	10·9	7·7	3–17	–	–
Front register	0·0	0·0	0–0	–	–
Low-calorie drinks					
All promotions	17·1	3·8	14–20	–	–
Endcaps	12·1	2·6	10–14	–	–
Front register	0·4	1·1	0–0	–	–
Juice					
All promotions	6·8	3·6	4–9	–	–
Endcaps	4·3	2·7	3–6	–	–
Front register	0·7	1·4	0–0	–	–

This table presents the mean, sd and interquartile range of the number of placement promotions per week in each food and beverage group, overall (i.e. at any location) and at endcaps and the front register. The table additionally presents the mean and sd for the proportion of promotions that were for healthier foods (i.e. received a rating of 1 or more stars by the Guiding Stars programme^(34,35)^), calculated as the average proportion of promoted items that were healthier across weeks. These values were calculated among foods that had non-missing healthfulness ratings (approximately 90 % of observations). There were no promotions at the front register for cold and frozen desserts. Additionally, the Guiding Stars programme did not rate the healthfulness of beverages at the time of the study. We therefore do not present information on the proportion of these promotions that were for healthier items.

When examining associations between in-store placement promotions and purchases, we found that households’ food group purchases generally did not change when they were exposed to a higher number of promotions for those food groups (Table 3). For example, a 1-sd increase in number of sweet and salty snack promotions (about 16 more promotions/week) was not associated with changes in households’ total purchases of sweet and salty snacks (B = –$0·10/week, 95 % CI –0·34, 0·14, adjusted *P* = 0·49), a pattern that was observed for promotions overall as well as for promotions at endcaps and the front register (Table 3). The exception to this pattern was that households purchased more candy during weeks with more promotions of candy on aisle endcaps (adjusted *P* < 0·001). This association was driven by non-participants in SNAP: a 1-sd increase in promotions of candy at aisle endcaps (about 1 more promotion/week over a mean of about 0·5 promotions/week) was associated with a $0·19 per week increase in purchases of candy among nonparticipants (95 % CI 0·09, 0·29, adjusted *P* < 0·001), with no similar association among SNAP participants (B = 0·09, 95 % CI –0·09, 0·26, adjusted *P* = 0·32).

**Table 3:** Associations between placement promotions and households’ food group purchases, overall and by participation in the Supplemental Nutrition Assistance Program (SNAP)

	Mean change in purchases per 1-sd increase in placement promotions								
	Overall, *n* 821			Households participating in SNAP, *n* 243			Households not participating in SNAP, *n* 578		
	Estimate	95 % CI	Adj. *P*	Estimate	95 % CI	Adj. *P*	Estimate	95 % CI	Adj. *P*
Foods									
Sweet and salty snacks									
All promotions	−0·10	–0·34, 0·14	0·49	0·22	–0·40, 0·83	0·49	−0·20	–0·47, 0·06	0·40
Endcaps	0·09	–0·13, 0·31	0·66	0·22	–0·23, 0·67	0·66	0·06	–0·20, 0·31	0·66
Front register	−0·12	–0·42, 0·18	0·43	0·56	–0·32, 1·45	0·43	−0·34	–0·63, –0·05	0·07
Baked goods									
All promotions	−0·01	–0·16, 0·14	0·89	−0·40	–0·87, 0·06	0·27	0·11	–0·04, 0·26	0·31
Endcaps	0·02	–0·14, 0·17	0·83	−0·33	–0·75, 0·09	0·27	0·12	–0·04, 0·28	0·27
Front register	−0·04	–0·14, 0·06	0·63	−0·29	–0·56, –0·01	0·12	0·02	–0·08, 0·13	0·63
Fruits, vegetables, beans and nuts									
All promotions	0·03	–0·36, 0·42	0·90	0·24	–0·61, 1·08	0·90	−0·03	–0·48, 0·43	0·90
Endcaps	−0·09	–0·38, 0·21	0·99	−0·33	–0·87, 0·21	0·70	0·00	–0·35, 0·35	0·99
Front register	−0·10	–0·64, 0·44	0·73	0·55	–1·08, 2·17	0·73	−0·28	–0·81, 0·26	0·73
Bread									
All promotions	0·00	–0·11, 0·11	0·98	0·00	–0·21, 0·21	0·98	0·00	–0·13, 0·13	0·98
Endcaps	−0·01	–0·12, 0·11	> 0·99	−0·02	–0·20, 0·15	> 0·99	0·00	–0·14, 0·14	> 0·99
Front register	0·03	–0·12, 0·18	0·87	−0·02	–0·29, 0·25	0·87	0·05	–0·13, 0·23	0·87
Packaged entrees and sides									
All promotions	0·04	–0·19, 0·27	0·71	0·33	–0·34, 0·99	0·71	−0·06	–0·28, 0·16	0·71
Endcaps	0·04	–0·18, 0·26	0·72	0·28	–0·36, 0·93	0·72	−0·04	–0·25, 0·17	0·72
Front register	0·07	–0·16, 0·29	0·71	0·43	–0·11, 0·96	0·35	−0·05	–0·30, 0·20	0·71
Cold and frozen desserts									
All promotions	0·02	–0·06, 0·09	0·81	0·02	–0·15, 0·20	0·81	0·02	–0·07, 0·10	0·81
Endcaps	0·02	–0·06, 0·09	0·81	0·02	–0·15, 0·19	0·81	0·02	–0·06, 0·10	0·81
Front register	–	––	–	–	––	–	–	––	–
Candy									
All promotions	0·03	–0·09, 0·16	0·58	−0·09	–0·29, 0·11	0·58	0·07	–0·08, 0·22	0·58
Endcaps	**0·16**	**0·07, 0·25**	**< 0·001**	0·09	–0·09, 0·26	0·32	**0·19**	**0·09, 0·29**	**< 0·001**
Front register	0·02	–0·10, 0·14	0·73	−0·11	–0·29, 0·07	0·65	0·06	–0·09, 0·20	0·73
Cereal									
All promotions	−0·02	–0·08, 0·05	0·74	−0·03	–0·18, 0·13	0·74	−0·02	–0·10, 0·06	0·74
Endcaps	0·01	–0·06, 0·08	0·80	−0·07	–0·20, 0·06	0·80	0·03	–0·05, 0·11	0·80
Front register	−0·01	–0·11, 0·08	0·76	0·12	–0·09, 0·32	0·56	−0·06	–0·17, 0·05	0·56
Beverages									
Sugar-sweetened beverages									
All promotions	−0·02	–0·19, 0·15	0·91	0·02	–0·37, 0·41	0·91	−0·03	–0·22, 0·16	0·91
Endcaps	−0·02	–0·17, 0·13	0·80	0·15	–0·27, 0·56	0·80	−0·07	–0·21, 0·08	0·80
Front register	0·02	–0·15, 0·19	0·81	−0·18	–0·59, 0·23	0·81	0·08	–0·11, 0·26	0·81
Unsweetened drinks									
All promotions	0·01	–0·08, 0·11	0·80	−0·02	–0·17, 0·13	0·80	0·02	–0·10, 0·13	0·80
Endcaps	0·00	–0·09, 0·09	0·98	0·04	–0·17, 0·25	0·98	−0·02	–0·12, 0·09	0·98
Front register	0·00	–0·09, 0·09	0·93	−0·03	–0·17, 0·10	0·93	0·02	–0·09, 0·13	0·93
Alcohol									
All promotions	0·03	–0·32, 0·38	0·88	0·31	–0·55, 1·17	0·88	−0·03	–0·43, 0·36	0·88
Endcaps	0·07	–0·19, 0·32	0·78	0·08	–0·45, 0·60	0·78	0·08	–0·22, 0·39	0·78
Front register	–	––	–	–	––	–	–	––	–
Low-calorie drinks									
All promotions	−0·01	–0·05, 0·02	0·49	0·03	–0·03, 0·09	0·49	−0·03	–0·07, 0·01	0·49
Endcaps	0·02	–0·02, 0·06	0·65	0·06	0·01, 0·11	0·05	0·01	–0·04, 0·05	0·84
Front register	−0·02	–0·06, 0·02	0·58	−0·03	–0·12, 0·07	0·58	−0·01	–0·05, 0·03	0·58
Juice									
All promotions	0·05	–0·01, 0·12	0·30	0·12	–0·04, 0·28	0·30	0·04	–0·04, 0·11	0·33
Endcaps	0·04	–0·01, 0·09	0·35	0·07	–0·03, 0·16	0·35	0·02	–0·04, 0·09	0·42
Front register	−0·03	–0·09, 0·04	0·41	0·08	–0·05, 0·22	0·41	−0·06	–0·13, 0·01	0·33

This table shows the associations between the number of weekly in-store placement promotions in a given food group and weekly purchases of that food group, overall and among participants and non-participants in the Supplemental Nutrition Assistance Program (SNAP). All models included household fixed effects and controlled for calendar month, calendar year, holiday weeks (weeks of Easter, Fourth of July, Halloween, Thanksgiving or Christmas), the average proportional discount applied to items purchased in that food group during that week (i.e. mean of discounts as a percentage of the item’s non-discounted price), intervention period (for the interventions implemented during the prior studies), the interaction between intervention period and intervention arm, SNAP issuance weeks in Maine (i.e. 10–14th of each month), whether the household used SNAP benefits for any transaction that week, and the interaction between SNAP issuance weeks and use of SNAP for any transaction that week. Models estimated robust standard errors clustered at the household level. Numbers of promotions were standardised prior to analyses so that estimates represent associations between a 1-sd (1-sd) increase in promotions and weekly purchases of each food group; standard deviations for weekly promotions are shown in Table 2. There were no cold and frozen dessert or alcohol promotions at the front register, so associations between front register promotions and purchases were not estimated for those categories. *P*-values were adjusted for multiple comparisons within food groups (three tests per group) by controlling the false discovery rate at *q* = 0·05 using Benjamini and Hochberg’s linear step-up method^(44)^; adjusted *P*-values are reported. Bold indicates association was statistically significant after correction for multiple comparisons, adjusted *P* < 0·05.

Results were similar when stratifying by product healthfulness. Households did not increase their overall purchases of healthier items from a given food group during weeks when they were exposed to greater in-store marketing of healthier products in that food group (Table 4). Likewise, a larger number of promotions of less healthy food group items was not associated with changes in households’ purchases of less healthy items from those food groups, regardless of food group.

**Table 4:** Associations between placement promotions and households’ food group purchases, by product healthfulness

	Healthier foods					Less healthy foods				
	Promotions per week		Mean change in purchases per 1-sd increase in placement promotions, $/week			Promotions per week		Mean change in purchases per 1-sd increase in placement promotions, $/week		
	Mean	sd	B	95 % CI	Adjusted *P*	Mean	sd	B	95 % CI	Adjusted *P*
Foods										
Sweet and salty snacks	40·7	9·3	0·00	–0·10, 0·10	0·97	77·4	8·8	−0·05	–0·17, 0·08	0·47
Baked goods	0·6	0·6	0·02	–0·01, 0·04	0·15	63·1	14·4	0·04	–0·07, 0·16	0·45
Fruits, vegetables, beans and nuts	23·8	10·1	0·13	–0·21, 0·47	0·47	12·7	4·7	−0·01	–0·07, 0·05	0·66
Bread	15·9	7·7	−0·01	–0·06, 0·04	0·69	17·3	6·1	−0·07	–0·16, 0·01	0·09
Packaged entrees and sides	3·7	2·7	−0·01	–0·04, 0·03	0·74	25·8	8·7	−0·05	–0·25, 0·15	0·64
Cold and frozen desserts	0·5	0·9	0·00	−0·004, 0·01	0·55	16·1	6·5	0·01	–0·06, 0·07	0·85
Candy	0·0	0·0	–	–	–	12·1	6·4	−0·04	–0·10, 0·02	0·24
Cereal	6·8	3·7	0·01	–0·03, 0·06	0·62	4·6	2·4	0·00	–0·04, 0·05	0·86
All foods	106·6	22·6	−0·01	–0·77, 0·76	0·99	299·0	32·4	−0·87	–2·06, 0·32	0·15

This table shows mean number of in-store placement promotions in each food category per week for healthier and less healthy items and the association between in-store promotions and weekly purchases of healthier and less healthy items in each food group. Items were classified as healthier if they received a rating of 1 or more stars by the Guiding Stars programme and as less healthy if they received a 0-star rating^(34,35)^. All models included household fixed effects and controlled for calendar month, calendar year, holiday weeks (weeks of Easter, Fourth of July, Halloween, Thanksgiving or Christmas), the average proportional discount applied to items purchased in that food group during that week, intervention period (for the interventions implemented during the prior studies), the interaction between intervention period and intervention arm, SNAP issuance weeks in Maine (i.e. 10–14th of each month), whether the household used SNAP benefits for any transaction that week, and the interaction between SNAP issuance weeks and use of SNAP for any transaction that week. Models estimated robust standard errors clustered at the household level. Numbers of promotions were standardised prior to analyses so that estimates represent associations between a 1-sd increase in promotions and weekly purchases of each food group. There were no promoted candy items categorised as healthier by Guiding Stars, so associations between promotions and purchases of healthier candy were not estimated. *P*-values were adjusted for multiple comparisons within food groups (two tests per group) by controlling the false discovery rate at *q* = 0·05 using Benjamini and Hochberg’s linear step-up method^(44)^.

## Discussion

In this longitudinal study, households with children were exposed to three times as many in-store placement promotions for less healthy foods compared to healthier foods. These households generally did not, however, increase their purchases of promoted food groups during weeks when they were exposed to a higher number of promotions of those food groups. The one exception was that endcap candy promotions were associated with higher candy purchases among non-participants in SNAP.

The households in this study were consistently exposed to a large number of promotions for less healthy foods and beverages. For example, during an average week, the study stores displayed approximately 131 sweet and salty snack promotions, 68 baked good promotions, 42 sugar-sweetened beverage promotions, 17 cold and frozen dessert promotions and 17 candy promotions; the vast majority of products promoted in these groups were less healthy. By contrast, during an average week, there were only thirty-eight promotions for fruits, vegetables, beans, and nuts, and thirteen promotions for cereals. The overall unhealthy nature of in-store marketing is concerning given that the average household shops for groceries multiple times per week^(45)^ and that children often accompany their parents to the grocery store^(46)^. Children therefore likely have considerable exposure to in-store promotions for less healthy foods. Given prior research showing that food marketing drives children’s food preferences^(4–8)^, our results suggest the need to identify strategies to improve the healthfulness of the retail food marketing environment. For example, grocery stores could make voluntary commitments to improve the healthfulness of the products they promote^(16,47)^, and policymakers could adopt policies that incentivise stores to place healthier products in prominent locations or restrict them from placing unhealthy products in these locations^(41,48)^.

After adjusting for multiple comparisons, the only association observed between in-store placement promotions and households’ food and beverage purchases was that a 1 promotion/week increase in candy promotions at aisle endcaps was associated with a modest increase in candy purchases (19 cents/week, an approximate 14·5 % increase over mean weekly candy purchases) among households not participating in SNAP. One potential explanation for why we observed associations for candy purchases but not for other food categories could be that candy is more likely to be an impulsive (*v*. planned) purchase^(49,50)^, potentially making candy purchases more responsive to increases in marketing compared to other food groups. Another possibility is that households were generally exposed to very few candy promotions on aisle endcaps (mean of 0·5 promotions/week), leaving more room for increases in endcap candy promotions to grab consumers’ attention and encourage them to buy candy. By contrast, households in this study were exposed to consistently high levels of promotions for other highly palatable foods like baked goods and sugary drinks. To our knowledge, only one other study has examined whether households’ purchases of an entire food group respond to greater in-store marketing of that food group. That study examined price promotions (not placement promotions) and found that when households are exposed to more promotions within a food group, they tend to buy slightly more items from that food group, with a stronger effect for less healthy product categories^(51)^. While that study did not examine the role of promotion location, our results suggest that aisle endcaps may be a particularly important location for driving up candy purchases.

Most associations between promotions and purchases were not statistically significant. It is worth noting, however, that this study examined natural variation in promotions that stores implemented as part of their normal operations, and for most of the less healthy food groups, there were few or no weeks when the number of promotions for that group was meaningfully low. For example, even the 25th percentile of number of weekly promotions for several food groups was relatively high: 118 promotions for sweet and salty snacks, 54 promotions for baked goods, and 32 promotions for sugar-sweetened beverages. It is possible that shoppers would respond more strongly to more substantial changes in marketing. Indeed, two studies found that eliminating placement of unhealthy foods at the checkout and nearby endcaps led to reductions in sales of the confectionery items often promoted in those locations^(15,52)^. Building on the success of those interventions, the United Kingdom recently implemented a policy to restrict placement of less healthy foods at checkouts, store entrances, and aisle endcaps^(43)^, and the city of Berkeley, CA has implemented a policy prohibiting stores from placing high-sugar or high-Na products at checkouts^(37)^. Future research should evaluate these strategies in US settings, including using randomised designs.

Strengths of this study include that we prospectively followed a cohort of households, analysed objectively measured purchases and studied households with children, who are an especially important population to target with healthy retail interventions. Limitations of this study include that both study supermarkets were located in the Northeast and the majority of participants identified as non-Hispanic White; studies in other areas and among more diverse populations are needed. Second, we were unable to examine how purchases of specific products changed when those products were promoted due to sparse data on purchases of any given product, though prior studies have addressed that question^(16–21)^. Instead, we examined purchases of entire food groups (e.g. sugar-sweetened beverages, candy, fruits and vegetables), outcomes that are likely more relevant for public health than purchases of a specific brand or product (e.g. Pepsi *v*. Coke). Third, although our data on in-store promotions likely captured most promotions, store managers may have implemented additional promotions (e.g. to promote locally sourced products), and these were not captured in our data. Fourth, although our analyses controlled for time-invariant household characteristics (e.g. stable preferences) to improve causal inference, there may be additional time-varying confounders.

### Conclusions

This longitudinal study of households with children found that the number of in-store placement promotions for food groups was not associated with greater total food group purchases, other than a modest increase in candy purchases in response to increased candy promotions on aisle endcaps. The lack of associations could be partially due to the unhealthy in-store marketing environment in the study supermarkets, which featured a consistently high number of promotions for less healthy foods and beverages and could have reduced our ability to detect the impact of additional promotions. Researchers and policymakers should explore interventions that make substantial improvements to the healthfulness of in-store food marketing.

## Supporting information

Grummon et al. supplementary material Grummon et al. supplementary material

## Supplementary material

For supplementary material accompanying this paper visit https://doi.org/10.1017/S1368980023002641
