# Supplementary material for: Effects of in-store marketing on food and beverage purchases: a longitudinal study of households with children
Source: Public Health Nutr. 2023 Dec 1;27(1):e4. doi: 10.1017/S1368980023002641 (PMC10830370; doi:10.1017/S1368980023002641)
Supplement: Grummon et al. supplementary material [file S1368980023002641sup001.docx]

**Effects of in-store marketing on food and beverage purchases: A longitudinal study of households with children**

**Online Supplemental Materials**

**Contents**

**Supplemental Methods.** Additional details on in-store promotion data

**Supplemental Figure 1**. Flowchart of exclusions

**Supplemental Table 1.** Food group definitions

**Supplemental References**

**Supplemental Methods.** Additional details on in-store promotion data

The following provides additional details on the in-store promotion data used in this study; see also Petimar et al.^1^

**1. Store types**

The chain’s corporate marketing team instructed stores that some products were to be promoted only in certain types of stores. The chain used three classification systems to group stores:

1. Theme stores: the chain indicated that some products were only to be promoted in “theme stores.” The chain’s corporate marketing team indicated which stores were “theme stores.”
2. Pallet stores: the chain indicated that some products were only to be promoted in supermarkets that were considered “pallet stores,” i.e., stores that sold products on wooden pallets. The chain’s corporate marketing team indicated which stores were “pallet stores.”
3. A,B,C,D,E stores: the study stores were classified by the chain as being an “A”, “B”, “C”, “D”, or “E” store, based on size. The marketing data specified whether products were promoted across all stores or only a subset based on this classification system.

**2. Determining the items promoted in each study store**

We used the chain’s marketing instructions alongside information on the physical attributes of the study stores to determine which products were promoted in each store. We linked data on mandated promotions to the store classification variables. For example, if the marketing data indicated that an item was promoted only in stores that were theme with classifications of A and B, then we only counted that item as promoted in stores that matched those criteria.

For items promoted on endcaps and front registers, we examined the number of endcaps and front registers in each store. The marketing data were directed toward all store managers, regardless of store size. Some stores had fewer endcaps and front registers than others, meaning they would not be able to promote all the items on the list of mandated promotions. The supermarket’s corporate marketing team therefore gave each item to be promoted at endcaps and front registers a priority number. Across all stores, items with priority number of 1 were to be placed at the store’s highest-selling endcap, those with a priority number of 2 were to be placed at the store’s 2^nd^ highest-selling endcap, etc. Stores with *x* endcaps thus only promoted items with priority numbers ≤ *x* (and same for front registers).

We determined which items were promoted at endcaps in each store by first calculating the number of endcaps in the store, which we calculated as the number of aisles in that store multiplied by 2 (i.e., each aisle had 1 endcap in the front of the aisle and 1 in the back). We then determined the number of “available endcaps” by subtracting 2 because store managers informed us that 1–2 endcaps in each store are typically reserved for special merchandising (e.g., gift cards) and cannot be used for chain-wide promotions. Then, an item was assumed to be promoted at the endcap if its priority number was less than the number of available endcaps in that store. For example, if a store had 8 aisles, we determined that it had 16 endcaps and 16-2=14 endcaps available for in-store promotions. For a given week, we assumed that this store only promoted items that had priority numbers 1-14 (i.e., it would not promote items designated by the chain to be on endcaps 15 or higher). We confirmed this matching strategy was accurate through conversations with store managers. We followed a similar approach for matching items to stores’ front registers (i.e., we assumed that a front register promotion was only implemented if the display number did not exceed the total number of front registers in a particular store).

**3. Examples of promotions**

The chain offered a variety of types of promotions, shown below.

| **Promotion type** | | **Photo example** |
| --- | --- | --- |
|  | |  |
| Endcaps | | 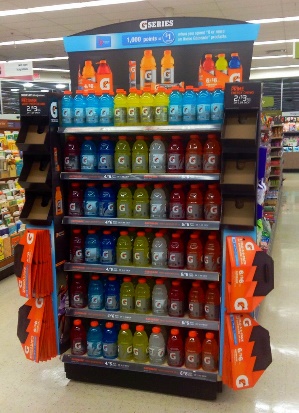 |
|  | |  |
| Front register | | 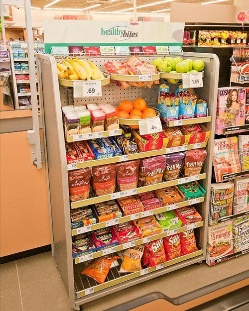 |
|  | |  |
| Theme displays | | 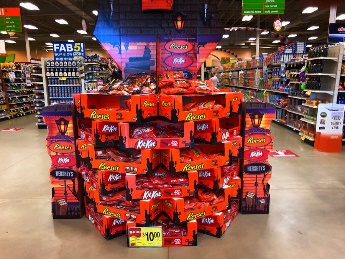 |
| Other | |  |
|  | Stack | 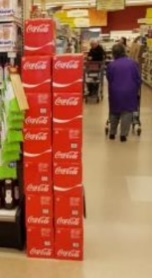 |
|  |  |  |
|  | Bin | 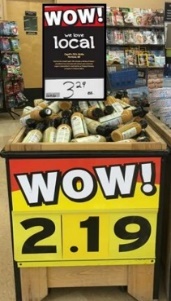 |
|  |  |  |
|  | Rack | 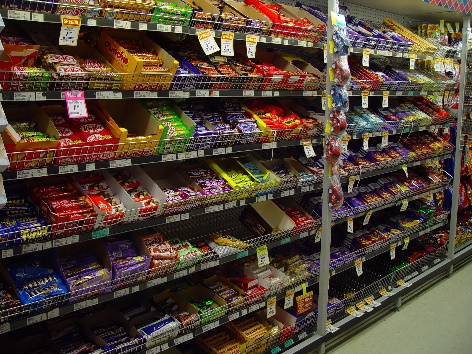 |
|  |  |  |
|  | Hutch | 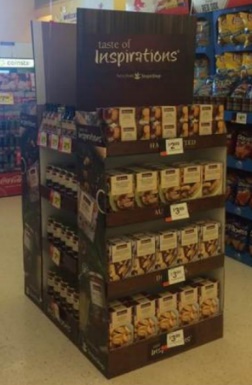 |
|  |  |  |
|  | Tiered display | 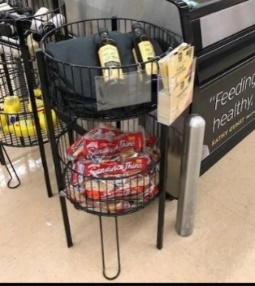 |

**4. Determining total number of promotions in each food group during each week**

We defined an individual promotion as a unique combination of signage, display, or both for a given UPC. For example, if a display at the front register included both a Coke product and a Pepsi product, we counted these as two sugar-sweetened beverage promotions.

**Supplemental Figure 1**. Flowchart of exclusions

**
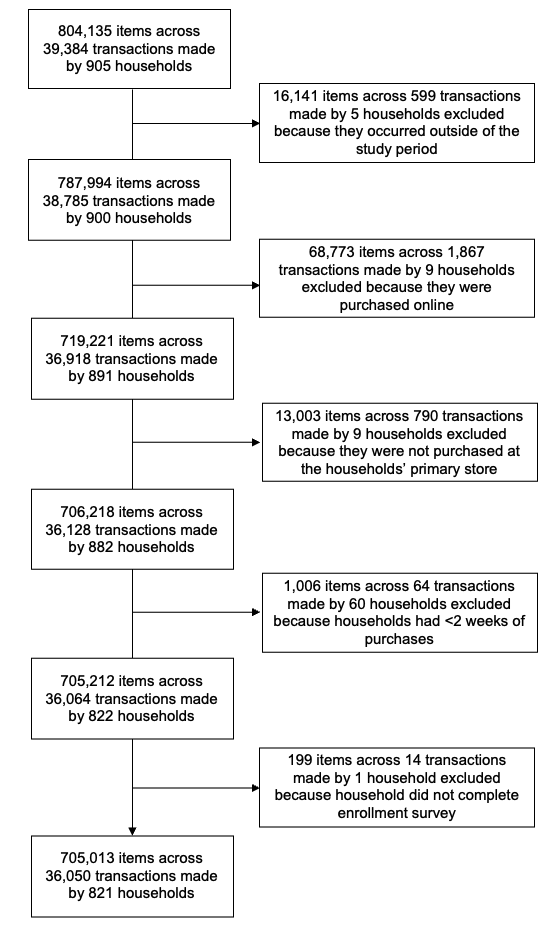
**

**Supplemental Table 1.** Food group definitions, see also Franckle et al.^2^

| **Food group** | **Definition** |
| --- | --- |
| **Foods** |  |
| Baked goods | Sweet breads, cakes, cinnamon rolls, croissants, Danishes, doughnuts, muffins, breakfast pastries, cookies, brownies, pies |
| Bread | Loaf breads, sandwich breads, thin breads, rolls, tortillas, taco shells, wraps, pita breads, specialty breads (e.g., brioche, baguettes), hamburger and hotdog buns, pizza crusts, bagels, English muffins |
| Candy | Candy, chocolate, gum, mints |
| Cereal | Ready-to-eat cereals, hot cereals (e.g., oatmeal, grits, farina), granola |
| Cold and frozen desserts | Ice cream, frozen yogurt, whipped cream, popsicles |
| Fruits, vegetables, beans, and nuts | Fresh, frozen, canned, or pureed fruits and vegetables, dried vegetables, 100% vegetable juice, black beans, black-eyed peas, chickpeas, kidney beans, lentils, lima beans, navy beans, pinto beans, soy beans, split beans, white beans, nuts, seeds, nut butters |
| Packaged entrees and sides | Packaged foods to be eaten away from the store (e.g., frozen, boxed, or canned meals, entrees, or sides) |
| Sweet and salty snacks | Chips, pretzels, popcorn, granola and cereal bars, dried fruit, crackers, snack mixes, pudding |
| **Beverages** |  |
| Alcohol | Alcoholic beverages intended for drinking, including beer, wine, and liquor |
| Juice | 100% fruit juice |
| Low-calorie drinks | Diet, light, or low-calorie versions of sugary drinks or fruit juice |
| Sugar-sweetened beverages | Any sugar-sweetened beverage including sodas, sports drinks, energy drinks, sweetened teas and coffees, fruit juices and drinks with added caloric sweetener, vegetable juice with added caloric sweetener, smoothies, hot chocolate, tonic water, bar mix, flavored milk or milk substitutes |
| Unsweetened drinks | Unflavored water, seltzer or soda water, coffee, tea, tea bags |

**References**

1. Petimar J, Moran AJ, Grummon AH, et al. In-Store Marketing and Supermarket Purchases: Associations Overall and by Transaction SNAP Status. *American Journal of Preventive Medicine*. Published online March 4, 2023. doi:10.1016/j.amepre.2023.02.029

2. Franckle RL, Moran A, Hou T, et al. Transactions at a northeastern supermarket chain: Differences by Supplemental Nutrition Assistance Program use. *Am J Prev Med*. 2017;53(4):e131-e138. doi:10.1016/j.amepre.2017.06.019
